# Generalized additive mixed model to evaluate the association between ventilatory ratio and mortality in patients: A retrospective cohort study

**DOI:** 10.1097/MD.0000000000040310

**Published:** 2024-11-01

**Authors:** Hongjie Yu, Jie Gu, Dang Lin

**Affiliations:** a Department of Thoracic Surgery, The Affiliated Suzhou Hospital of Nanjing Medical University, Suzhou Municipal Hospital, Suzhou, China; b Department of Respiratory Medicine, The Affiliated Suzhou Hospital of Nanjing Medical University, Suzhou Municipal Hospital, Suzhou, Jiangsu, China.

**Keywords:** 30-day mortality, 90-day mortality, ventilatory ratio

## Abstract

Previous studies have indicated that a higher ventilatory ratio (VR) is associated with mortality. However, it is unknown whether dynamic changes in VR over time affect the prognosis of critically ill patients. This study aims to investigate the significance of VR during the progression of the disease in critically ill patients. The Medical Information Mart for Intensive Care III database was searched to identify critically ill patients. The primary outcome was 30-day mortality. Multivariable Cox regression was used to elucidate the relationship between the VR and mortality. Finally, we employed a generalized additive mixed model to compare trends in VR over time between survivors and non-survivors. A total of 8024 patients were enrolled. Multivariable Cox regression analysis identified a baseline VR ≥1.89 as an independent risk factor predicting 30-day mortality (hazard ratio: 2.10, 95% confidence interval: 1.89–2.33, *P* < .001) and 90-day mortality (hazard ratio: 2.18, 95% confidence interval: 1.97–2.41, *P* < .001) after adjusting for potential confounders. In the subgroup analyses, the observed association between VR and 30-day mortality showed consistent direction across most subgroups. The generalized additive mixed model results highlighted that the difference in VR between survivors and non-survivors increased by an average of 0.01 per day after adjusting for several covariates. In conclusion, VR dynamically mirrors pathophysiological changes in critically ill patients and its escalation is linked to higher mortality rates. Monitoring VR’s dynamic shifts might offer more immediate prognostic information, thus aiding in timely interventions and risk stratification.

## 1. Introduction

The quantification of pulmonary dead space – represented by the dead space/tidal volume ratio – reflects that fraction of each breath’s tidal volume which fails to participate in the elimination of carbon dioxide. It serves as a robust overall indicator of the lungs’ functional efficacy and correlates with mortality rates among patients in critical condition.^[1,2]^ Despite its significance, the Berlin Definition did not incorporate pulmonary dead space due to the requirement of specialized equipment for its assessment, which rendered it infrequently utilized within everyday clinical settings, thus constraining its practicality.

In response to these limitations, the ventilatory ratio (VR) has emerged as a viable and accessible surrogate marker for dead space. It is calculated as [ventilation per minute (mL/min) × PaCO_2_ (mm Hg)]/[predicted body weight (kg) × 100 × 37.5]. This index offers a simplified yet effective bedside measure of ventilatory efficiency.^[3]^ Furthermore, VR has been substantiated as an independent prognostic factor for mortality among critically ill patients, reinforcing its clinical relevance.^[4,5]^ Investigations have also highlighted VR’s potential in anticipating the likelihood of extubation failure.^[6]^ However, it remained unclear if VR’s prognostic value persisted across temporal changes, as variations in test outcomes could potentially reflect a patient’s clinical improvement posttreatment. To elucidate this, we applied a generalized additive mixed model (GAMM) to analyze the dynamic patterns of VR in relation to disease progression in a critically ill cohort.

## 2. Data source

We conducted a retrospective single-center study based on a large US-based database called the Medical Information Mart for Intensive Care III (MIMIC-III),^[7]^ which contains data associated with over 50,000 distinct intensive care unit (ICU) hospital patients between 2001 and 2012. The MIMIC-III (v1.4) database contains comprehensive and high-quality data on well-defined and characterized patients admitted to ICUs at the Beth Israel Deaconess Medical Center. The institutional review boards of the Massachusetts Institute of Technology (Cambridge, MA) and Beth Israel Deaconess Medical Center (Boston, MA) approved the establishment of the database. The database is accessible to researchers who have completed protecting human subjects training. The data presented in this study were extracted by author Gu, who completed the online training course from the the National Institutes of Health (certification number: 34397689).

## 3. Population selection criteria

Eligible patients were those who were over 18 years old at admission. Patients were excluded from our study based on the following criteria: more than 5% of their individual data were missing; absence of data on VR at the first admission; baseline values exceeding the mean ± 3 standard deviations. Additionally, we only analyzed the initial ICU stay for patients who had multiple admissions to the ICU.

## 4. Data extraction

We chose 30-day mortality as the primary outcome for this study. Ninety-day mortality was the secondary outcome. Patient data were extracted from MIMIC-III using Structured Query Language with PostgreSQL tools. The extracted data included age, gender, care unit, and severity at admission as measured by Sequential Organ Failure Assessment (SOFA) score and the Simplified Acute Physiology Score II. Comorbidities included cirrhosis, chronic obstructive pulmonary disease (COPD), diabetes, sepsis, hypertension, congestive heart failure (CHF), and chronic kidney disease (CKD). Vital signs included the mean arterial pressure, heart rate, temperature, and respiratory rate. Laboratory variables including white blood cell count, platelet count, creatinine, lactate, and albumin were measured during the first 24 hours after admission. Use of vasopressors and oxygenation index (OI) were measured. We estimated VR as [minute ventilation (mL/min) × arterial partial tension of carbon dioxide (mm Hg)]/[predicted body weight × 100 × 37.5].^[3]^ Repeated measurements of VR for each patient were conducted during the 30 days following admission.

## 5. Statistical analysis

Continuous variables are represented as mean ± standard deviation or median (25th quartile, 75th quartile). The *t* test was applicable for continuous variables with a normality and homogeneous variance. Alternatively, the Wilcoxon test was applicable. The categorical variables were presented as percentages, and the *X*^2^ test was appropriate.

We divided the patients into 2 groups based on the cutoff determined by maximally selected rank statistics of patients.^[8]^ Then, we utilized Kaplan–Meier survival analysis to compare the 30 and 90-day mortality between the 2 groups.

Multivariable Cox regression was conducted to assess the relationship between mortality and VR. Model 1 adjusted for common confounding variables, including age and gender. Model 2 was the full model, with adjusted variables for age, gender, cirrhosis, COPD, diabetes, sepsis, hypertension, CHF, CKD, SOFA score, SAPS II score, vasopressors, and OI. Before applying the model, variable inflation factors (VIF) are used to test multicollinearity for each group of the independent variables. We remove the variables with a VIF above 10.

Subgroup analyses were conducted to assess potential variations in the efficacy of the VR among different subgroups stratified by age, gender, SOFA, SAPS II, use of vasopressors, OI, and comorbidities (such as cirrhosis, sepsis, COPD, hypertension, CHF, CKD, and ARDS). Propensity score matching (PSM) was employed to control potential confounders and create comparable patient groups for the high-VR group and low-VR group.

Restricted cubic spline with 3 knots was adopted to visualize the potentially nonlinear association between the VR and 30-day mortality.^[9]^

In this study, longitudinal data were the VR over time. The longitudinal VR was analyzed using the GAMM, which easily accommodates unbalanced and unequally spaced observations. This makes it an ideal tool for analyzing longitudinal data.^[10–12]^ All models also included intercept and time as random factors. In the mixed-effects model, the interaction term between a fixed effect variable and time assesses whether this variable predicts the longitudinal changes in the VR.

A 2-tailed *P* value <.05 was deemed statistically significant. All analyses were performed with the R software (version 4.2.3).

## 6. Results

### 6.1. Subject characteristics

**Figure 1.**
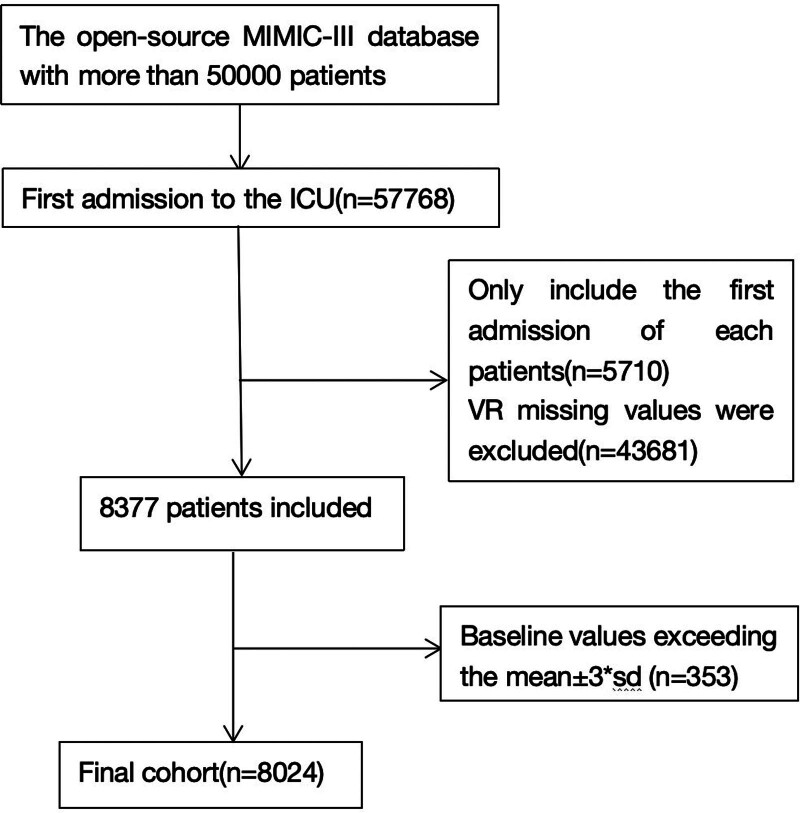
Study flow diagram in the present study.

We initiated our investigation by accessing the medical records of over 40,000 individuals admitted to the ICU at Beth Israel Deaconess Medical Center, sourced from the MIMIC-III database. Post-application of our predefined inclusion and exclusion criteria, a cohort of 8024 participants (illustrated in Fig. 1) met the eligibility requirements for our study.

**Figure 2.**
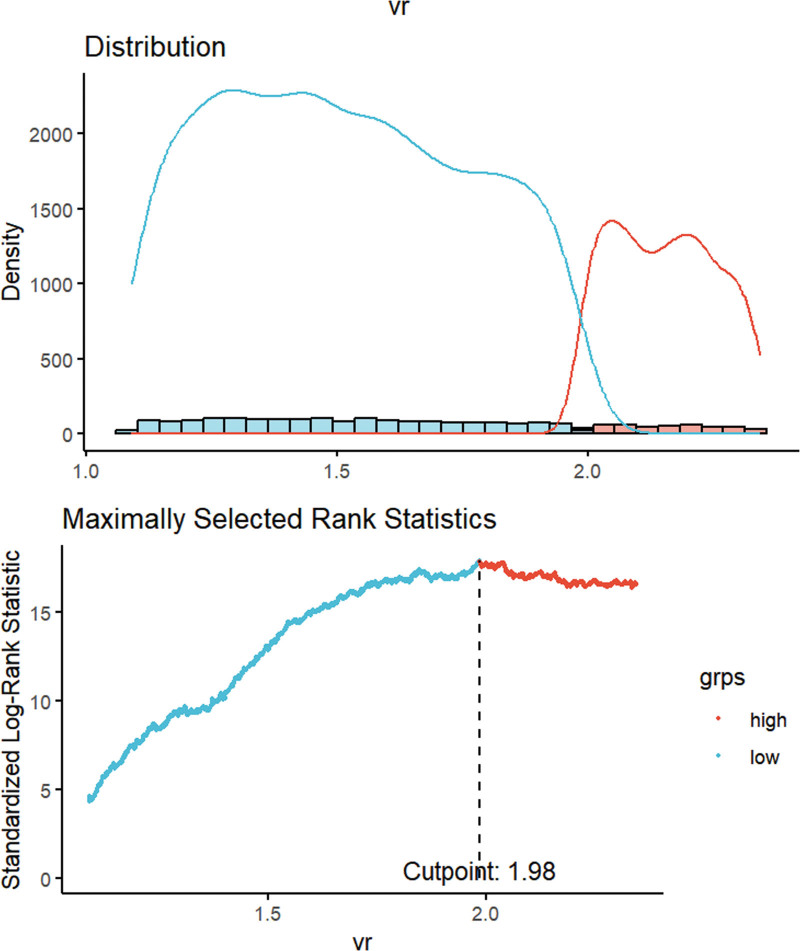
The cutoff point was calculated using the maximally selected rank statistics based on the “maxstat” package. SLRS = Standardized Log-Rank Statistic.

We stratified the patients into 2 cohorts based on a threshold VR value determined by maximally selected rank statistics (Fig. 2). The cohort with a low-VR (VR < 1.89) comprised 76.5% of the study population (n = 6140), while the high-VR group (VR ≥ 1.89) constituted the remaining 23.5% (n = 1884). We detailed the demographic and clinical characteristics of these groups in Table 1, revealing a higher proportion of males and higher prevalence of COPD and sepsis within the high-VR cohort. Additionally, the high-VR group presented elevated SOFA and SAPS II scores compared to their low-VR counterparts.

**Table 1:** Baseline characteristics of this study population.

Variables	Ventilatory ratio		*P* value
	<1.89 (n = 6140)	≥1.89 (n = 1884)	
Age	70.65 (41.29)	70.51 (45.71)	.907
Gender	2063 (33.6)	995 (52.8)	<.001
Care unit			<.001
CCU	566 (9.2)	277 (14.7)	
CSRU	3409 (55.5)	488 (25.9)	
MICU	863 (14.1)	666 (35.4)	
SICU	823 (13.4)	273 (14.5)	
TSICU	479 (7.8)	180 (9.6)	
Comorbidities			
Cirrhosis	271 (4.4)	84 (4.5)	.934
COPD	73 (1.2)	150 (8)	<.001
Diabetes	1828 (29.8)	559 (29.7)	.933
Hypertension	3327 (54.2)	824 (43.7)	<.001
CHF	3086 (50.3)	616 (32.7)	<.001
CKD	3086 (50.3)	616 (32.7)	<.001
Sepsis	570 (9.3)	377 (20)	<.001
Vital signs			
MAP (mm Hg)	77 (71–82)	73 (67–82)	<.001
Heart rate (bpm)	86 (78–94)	91 (79–105)	<.001
Respiratory rate (bpm)	17 (15–20)	20 (17–24)	<.001
Temperature (°C)	36.9 (36.6–37.3)	36.8 (36.2–37.4)	<.001
Laboratory tests			
WBC (×10^9^/L)	11.6 (8.6–15.4)	13.3 (8.9–18.6)	<.001
Platelet (×10^9^/L)	176 (126–244)	191 (123–267)	.043
Creatinine (mg/dL)	0.9 (0.7–1.2)	1.3 (0.8–2.1)	<.001
Lactate level (mmol/L)	1.7 (1.2–2.7)	2.4 (1.5–4.8)	<.001
Albumin (g/dL)	2.8 (2.4–3.3	2.6 (2.2–3.2)	<.001
Severity scale			
SOFA	5 (3–7)	6 (4–8)	<.001
SAPS II	35 (28–44.2)	40 (31–51)	<.001
Vasopressor use	337 (5.5)	300 (15.9)	<.001
OI	355.31 (219.38)	243.45 (214.02)	<.001
Outcome			
90-d mortality	615 (10.0)	515 (27.3)	<.001
30-d mortality	528 (8.6)	486 (25.8)	<.001
LOS	3 (1.5–5.9)	5.2 (2.2–12.8)	<.001

CCU = coronary care unit, CHF = congestive heart failure, CKD = chronic kidney disease, COPD = chronic obstructive pulmonary disease, CSRU = cardiac surgery unit, MAP = mean arterial pressure, MICU = medical intensive care, OI = oxygenation Index, LOS = length of stay, SAPS II = simplified acute physiology score II, SICU = surgical intensive care unit, SOFA = Sequential Organ Failure Assessment, TSICU = trauma surgical intensive care unit, WBC = white blood cell.

### 6.2. Association between VR and 30 and 90-day mortality

Our analysis compared survival outcomes between high-VR and low-VR groups. Kaplan–Meier survival plots for 30 and 90-day mortality revealed that the low-VR cohort had significantly better cumulative survival rates (log-rank test, *P* < .001), a pattern consistent for both 30 and 90-day observations (eFigure 1, Supplemental Digital Content, http://links.lww.com/MD/N831).

**Figure 3.**
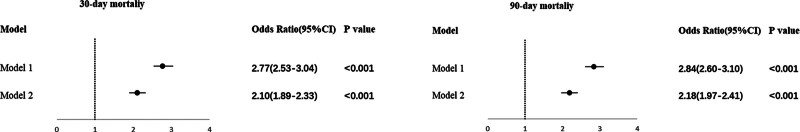
Cox proportional hazard models exploring the association of VR with 30 and 90-d mortality. CI = confidence interval, VR = ventilatory ratio.

We further explored VR’s impact on survival through Cox proportional hazards modeling for both 30 and 90-day mortality outcomes. In model I, which adjusted for age and gender, a high-VR (VR ≥ 1.89) independently predicted an increased risk of mortality at both time points (both *P *< .001), with hazard ratios (HR) of 2.77 (95% confidence interval [CI]: 2.60–3.10) for 30-day mortality and 2.84 (95% CI: 2.60–3.10) for 90-day mortality. In model II, even after extensive adjustment for comorbidities and clinical severity scores, high-VR remained a significant predictor of mortality (HR for 30-day mortality: 2.10 [95% CI: 1.89–2.33]; HR for 90-day mortality: 2.18 [95% CI: 1.97–2.41]; both *P* < .001) (depicted in Fig. 3). The collinearity diagnostic analysis showed that the VIFs of those risk factors were <10, suggesting that there is no strong indication of multicollinearity among variables (eFigure 2, Supplemental Digital Content, http://links.lww.com/MD/N831).

In subgroup analyses, the association between VR and 30-day mortality risk remained significant across most groups (*P* < .05), with the exception of patients with COPD (detailed in eFigure 3, Supplemental Digital Content, http://links.lww.com/MD/N831).

**Figure 4.**
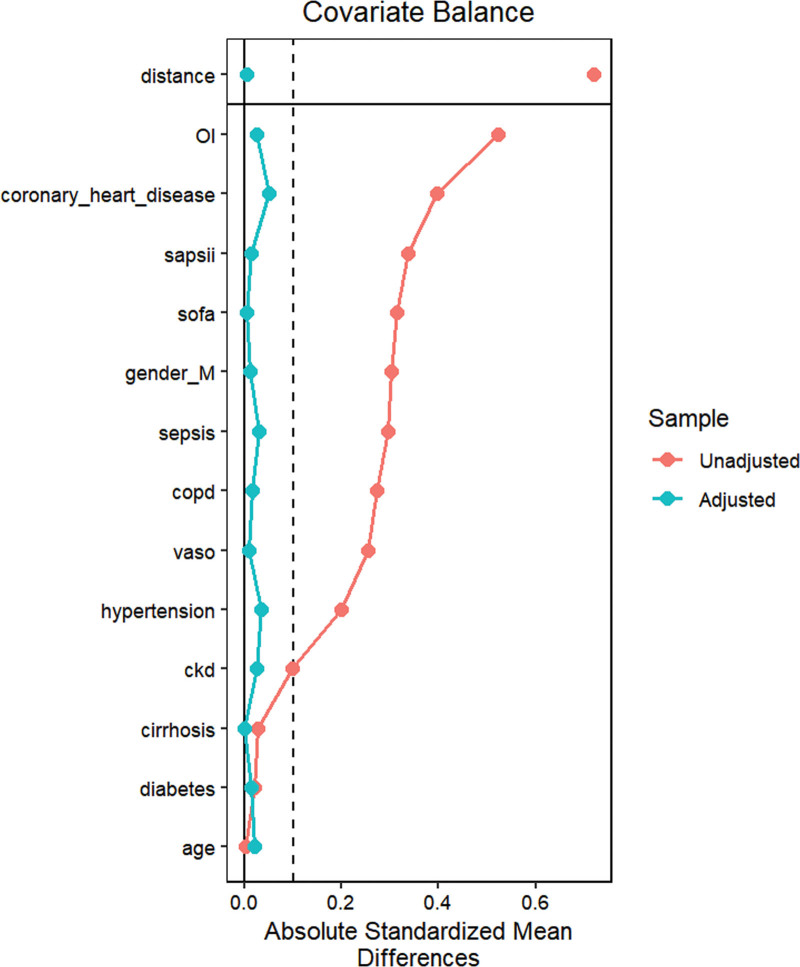
The absolute standardized differences for the matching variables between the 2 groups. CKD = chronic kidney disease, COPD = chronic obstructive pulmonary disease, OI = oxygenation index.

To mitigate baseline characteristic disparities between low-VR and high-VR groups, a 1:1 PSM strategy was employed, culminating in the pairing of 1694 patient dyads. Subsequent to PSM, a congruence in demographics, comorbidities, severity scale, and administered treatments was observed between the cohorts, as outlined in Table 2. The efficacy of the PSM was evaluated both pre- and post-PSM, with these results depicted in Figure 4. Following PSM, discernible disparities remained between the cohorts at various timeframes: 90-day mortality (19.2% vs 26.3%, *P* < .001), 30-day mortality (16.6% vs 23.8%, *P* < .001), LOS (3.96 vs 4.77, *P* < .001).

**Table 2:** Baseline characteristic after PSM.

Variables	Ventilatory ratio		*P* value
	<1.89 (n = 1694)	≥1.89 (n = 1694)	
Age	71.61 (44.14)	70.63 (46.59)	.034
Gender	872 (51.5)	863 (50.9)	.783
Comorbidities			
Cirrhosis	73 (4.3)	73 (4.3)	1.000
COPD	62 (3.7)	70 (4.1)	.534
Diabetes	524 (30.9)	514 (30.3)	.737
Hypertension	723 (42.7)	752 (44.4)	.332
CHF	516 (30.5)	555 (32.8)	.160
CKD	271 (16.0)	256 (15.1)	.507
Sepsis	345 (20.4)	324 (19.1)	.338
Severity scale			
SOFA	5 (4–8)	6 (4–8)	.664
SAPS II	40 (31–51)	40 (31–51)	.569
Vasopressor use	235 (13.9)	230 (13.6)	.842
OI	265.74 (197.44)	251.07 (218.36)	.040
Outcome			
90-d mortality	326 (19.2)	446 (26.3)	<.001
30-d mortality	282 (16.6)	404 (23.8)	<.001
LOS	3.96 (2.04–8.96)	4.77 (2.19–10.90)	<.001

CHF = congestive heart failure, CKD = chronic kidney disease, COPD = chronic obstructive pulmonary disease, LOS = length of stay, OI = oxygenation Index, PSM = propensity score matching, SAPS II = simplified acute physiology score II, SOFA = Sequential Organ Failure Assessment.

**Figure 5.**
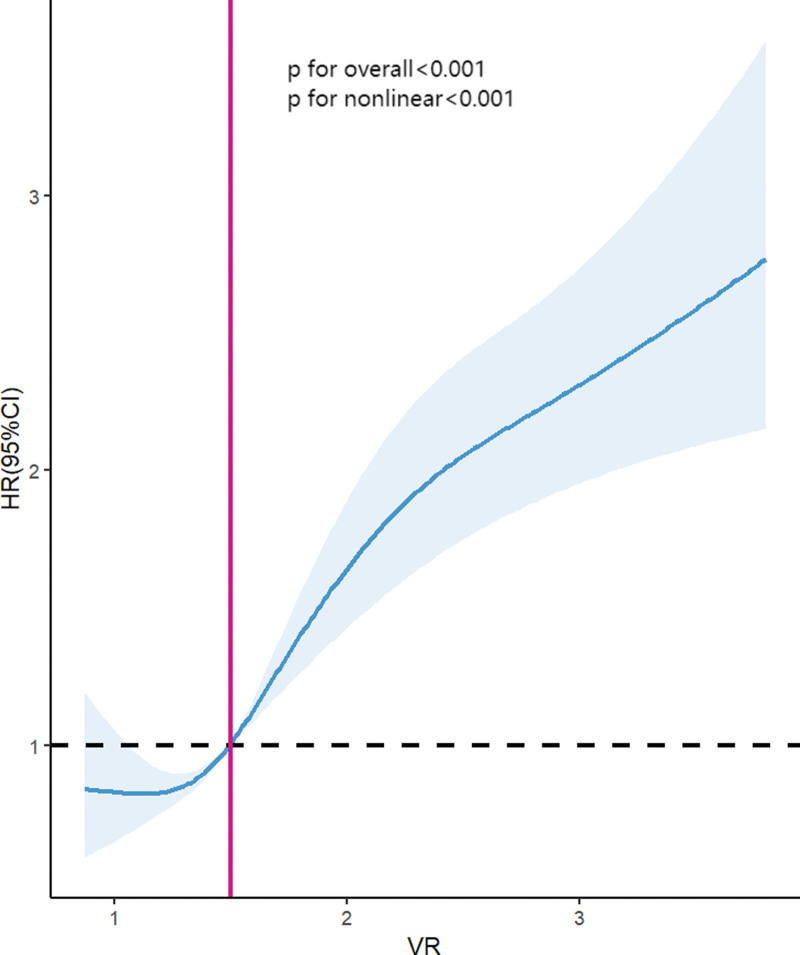
The association of VR with 30-d mortality by restricted cubic spline. HRs were adjusted for age, gender, cirrhosis, COPD, diabetes, sepsis, hypertension, CHF, CKD, SOFA, SAPS II, vasopressors, and OI. CHF = congestive heart failure, CKD = chronic kidney disease, COPD = chronic obstructive pulmonary disease, HR = hazard ratio, OI = oxygenation index, SAPS II = Simplified Acute Physiology Score II, SOFA = Sequential Organ Failure Assessment, VR = ventilatory ratio.

In Figure 5, a nonlinear association of VR with 30-day mortality was demonstrated on a continuous scale with restricted cubic spline curved based on Cox proportional hazards models (both *P* for nonlinear <.001, both *P* for overall <.001).

### 6.3. Association between changes in VR and 30-day mortality

**Figure 6.**
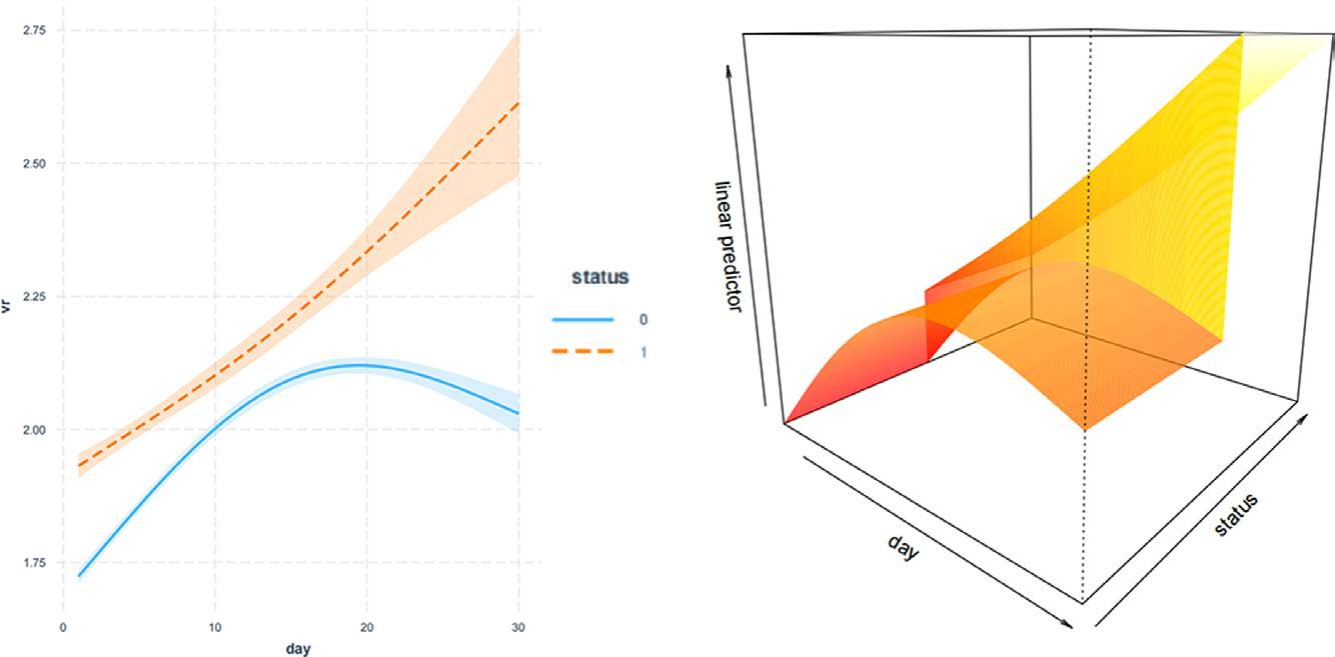
Association between dynamic change in VR over time and 30-d mortality. A nonlinear relationship was found between changes in VR over time and 30-d mortality by GAMM. The adjusted covariates include age, gender, cirrhosis, COPD, diabetes, sepsis, hypertension, CHF, CKD, SOFA, SAPS II, vasopressors, and OI. CHF = congestive heart failure, CKD = chronic kidney disease, COPD = chronic obstructive pulmonary disease, GAMM = generalized additive mixed model, OI = oxygenation index, SAPS II = Simplified Acute Physiology Score II, SOFA = Sequential Organ Failure Assessment, VR = ventilatory ratio.

We distinguished VR trends across various time intervals by comparing 30-day survivors to non-survivors. VR disparities were significant across multiple time points ranging from admission through to the 30th day (presented in eTable 1, Supplemental Digital Content, http://links.lww.com/MD/N832). Employing GAMM to adjust for confounding factors (age, gender, cirrhosis, COPD, diabetes, sepsis, hypertension, CHF, CKD, SOFA score, SAPS II score, vasopressors, and OI), we noted a pattern wherein VR levels peaked and stabilized thereafter among survivors. Conversely, in the non-survivor group, VR progressively increased. A focused comparison between survivors and non-survivors facilitated a deeper understanding of the relation between VR trajectory and 30-day mortality (illustrated in Fig. 6). Notably, Table 3 captures a significant divergence in VR trends between the 2 groups during this period, with an average daily increase of 0.01. This increment persisted at 0.01 daily after adjusting for covariates, reinforcing the robustness of these findings.

**Table 3:** Relationship between changes in VR and 30-d mortality in patients derived from a linear mixed-effects regression model.

Outcomes	Model 1		Model 2	
	B (95% CI)	*P* value	B (95% CI)	*P* value
Intercept	1.72 (1.70–1.75)	<.001	1.70 (1.66–1.74)	<.001
Day	0.02 (0.01–0.02)	<.001	0.01 (0.01–0.01)	<.001
Death	0.28 (0.24–0.31)	<.001	0.21 (0.17–0.24)	<.001
Day × death	0.01 (0.00–0.01)	.035	0.01 (0.00–0.01)	.004

Model 1 adjusted for common confounding variables, including age and gender. Model 2 was the full model, with adjusted variables for age, gender, cirrhosis, COPD, diabetes, sepsis, hypertension, CHF, CKD, SOFA score, SAPS II score, vasopressors, and OI.

CHF = congestive heart failure, CI = confidence interval, CKD = chronic kidney disease, COPD = obstructive pulmonary disease, OI = oxygenation index, SAPS II = Simplified Acute Physiology Score II, SOFA = Sequential Organ Failure Assessment, VR = ventilatory ratio.

## 7. Discussion

Our retrospective study delved into the dynamic shifts in the VR as a predictive marker for mortality in a general ICU patient population. The analysis utilizing Cox proportional hazards models unearthed a notable independent correlation between VR and both 30 and 90-day mortality. Furthermore, results from the GAMM underscored significant variations in VR between survivors and non-survivors during the 10 to 30 days following admission, with these associations remaining robust even after confounder adjustment. The discrepancy in VR between the 2 cohorts increased daily by 0.018 within that timeframe. Consequently, continuous VR monitoring may provide crucial insights into identifying patients at an elevated risk of adverse outcomes.

The VR, a relatively recent innovation for the bedside evaluation of ventilatory efficiency, mirrors physiological dead space and has been acknowledged as a mortality predictor in ARDS patients.^[13]^ Its association with mortality has been substantiated in robust clinical studies and observational cohorts involving ARDS.^[14]^ Moreover, VR proved to be a more potent mortality predictor in intubated coronavirus disease 2019 patients in 1 study.^[15]^ Monteiro et al^[16]^ and Sinha et al^[14]^ demonstrated that patients with VR >2 (median) on day 1 had significantly lower 90-day survival than those with VR ≤2. Consistent with our findings, other research has indicated that a higher VR (≥1.89) correlates with increased mortality risk in critically ill patients.

However, these studies only focused on the correlation between baseline VR and mortality. Yet, it was unknown whether VR maintained its predictive value in tracking evolution over time, as changes in test results may indicate whether the patient has improved after treatment. Therefore, we utilized the GAMM model to examine the relationship between temporal changes in VR and mortality. Several studies have reported that baseline VR on the first day was significantly associated with a higher risk of mortality.^[5,14,17]^ Monteiro et al^[16]^ demonstrated that the VR was significantly different between survivors and non-survivors on the second day.^[18]^ Previous literature has often limited its scope to the initial week post-admission. In a secondary analysis that tracked the trajectory of VR up to day 21,^[19]^ it was observed that there was an upward trend in VR up to day 7, followed by a plateau up to day 21 in both non-survivors and survivors. We extended the follow-up period in our study and found that both the survival and non-survival groups showed a gradual increase in VR over the course of the early stage. The increase in the non-survival group was significantly greater compared to that of the survival group in the later stage, even after adjusting for potential confounders. We observed a progressive widening of the gap in the increase of VR between the 2 groups (an average of 0.018 per day) during the 10 to 30 days after admission.

The extent to which VR-mortality associations vary according to baseline illness severity remains an open question. Through subgroup analyses, we confirmed the direction and robustness of the VR and 30-day mortality association across most subgroups.

Several limitations should be considered in the present study. The retrospective design may introduce selection bias, and future prospective studies are encouraged to validate our results. Moreover, the random timing of VR measurements, with inconsistent data availability across patients, could affect the findings. Additionally, given that the MIMIC-III dataset spans from 2001 to 2015, changes in clinical practice over time might influence our results. Hence, longitudinal studies with repeated VR measures could further elucidate this relationship. Therefore, a longitudinal study design with repeated measures of VR might be valuable in delineating this relationship further.

## 8. Conclusions

In conclusion, VR dynamically mirrors pathophysiological changes in critically ill patients and its escalation is linked to higher mortality rates. Monitoring VR’s dynamic shifts might offer more immediate prognostic information, thus aiding in timely interventions and risk stratification.

## Author contributions

**Data curation:** Hongjie Yu.

**Writing**—**original draft:** Hongjie Yu.

**Formal analysis:** Jie Gu.

**Investigation:** Jie Gu.

**Methodology:** Jie Gu.

**Software:** Jie Gu.

**Conceptualization:** Dang Lin.

**Supervision:** Dang Lin.

**Writing**—**review & editing:** Dang Lin.

## Supplementary Material



## References

[1] Cepkova M Kapur V Ren X. Pulmonary dead space fraction and pulmonary artery systolic pressure as early predictors of clinical outcome in acute lung injury. Chest. 2007;132:836–42. 17573490 10.1378/chest.07-0409

[2] Siddiki H Kojicic M Li G. Bedside quantification of dead-space fraction using routine clinical data in patients with acute lung injury: secondary analysis of two prospective trials. Crit Care. 2010;14:R141. 20670411 10.1186/cc9206 PMC2945122

[3] Sinha P Fauvel NJ Singh S Soni N. Ventilatory ratio: a simple bedside measure of ventilation. Br J Anaesth. 2009;102:692–7. 19346233 10.1093/bja/aep054

[4] Sinha P Singh S Hardman JG Bersten AD Soni N. Evaluation of the physiological properties of ventilatory ratio in a computational cardiopulmonary model and its clinical application in an acute respiratory distress syndrome population. Br J Anaesth. 2014;112:96–101. 24067330 10.1093/bja/aet283 PMC9585654

[5] Torres A Motos A Riera J; CIBERESUCICOVID Project (COV20/00110, ISCIII). The evolution of the ventilatory ratio is a prognostic factor in mechanically ventilated COVID-19 ARDS patients. Crit Care. 2021;25:331–413. 34517881 10.1186/s13054-021-03727-x PMC8436582

[6] Yang H Ni Y Huang D Liang Z. Ventilatory ratio as a predictor for extubation failure in critical ill patients based on MIMIC-IV database (from 2008 to 2019). Front Physiol. 2023;14:1137115. 37324397 10.3389/fphys.2023.1137115 PMC10267390

[7] Johnson AE Pollard TJ Shen L. MIMIC-III, a freely accessible critical care database. Sci Data. 2016;3:160035. 27219127 10.1038/sdata.2016.35 PMC4878278

[8] Zhang L Chen S Wang W Wang Y Liang Y. Inflammatory and nutritional scoring system for predicting prognosis in patients with newly diagnosed multiple myeloma. J Infamm Res. 2023;16:7–17. 10.2147/JIR.S390279 PMC9831084 36636247

[9] Harre FE Lee KL Pollock BG. Regression models in clinical studies: determining relationships between predictors and response. J Natl Cancer Inst. 1988;80:1198–202. 3047407 10.1093/jnci/80.15.1198

[10] Hartl WH Kopper P Bender A. Protein intake and outcome of critically ill patients: analysis of a large international database using piece-wise exponential additive mixed models. Crit Care. 2022;26:7. 35012618 10.1186/s13054-021-03870-5 PMC8751086

[11] Ramjith J Roes KCB Zar HJ Jonker MA. Flexible modelling of risk factors on the incidence of pneumonia in young children in South Africa using piece-wise exponential additive mixed modelling. BMC Med Res Methodol. 2021;21:17. 33430789 10.1186/s12874-020-01194-6 PMC7802241

[12] Zhang T Li X Ji X Lu J Fang X Bian Y. Generalized additive mixed model to evaluate the association between total pulmonary infection volume and volume ratio, and clinical types, in patients with COVID-19 pneumonia: a propensity score analysis. Eur Radiol. 2021;31:7342–52. 33855587 10.1007/s00330-021-07860-7 PMC8046497

[13] Nuckton TJ Alonso JA Kallet RH. Pulmonary dead-space fraction as a risk factor for death in the acute respiratory distress syndrome. N Engl J Med. 2002;346:1281–6. 11973365 10.1056/NEJMoa012835

[14] Sinha P Calfee CS Beitler JR. Physiologic analysis and clinical performance of the ventilatory ratio in acute respiratory distress syndrome. Am J Respir Crit Care Med. 2019;199:333–41. 30211618 10.1164/rccm.201804-0692OC PMC6363976

[15] Bos LDJ Sjoding M Sinha P; PRoVENT-COVID Collaborative Group. Longitudinal respiratory subphenotypes in patients with COVID-19-related acute respiratory distress syndrome: results from three observational cohorts. Lancet Respir Med. 2021;9:1377–86. 34653374 10.1016/S2213-2600(21)00365-9 PMC8510633

[16] Monteiro ACC Vangala S Wick KD; NHLBI PETAL Network. The prognostic value of early measures of the ventilatory ratio in the ARDS ROSE trial. Crit Care. 2022;26:297.5. 36175982 10.1186/s13054-022-04179-7 PMC9521854

[17] Ende VJ Singh G Babatsikos I. Survival of COVID-19 patients with respiratory failure is related to temporal changes in gas exchange and mechanical ventilation. J Intensive Care Med. 2021;36:1209–16. 34397301 10.1177/08850666211033836 PMC8442134

[18] Morales-Quinteros L Schultz MJ Bringué J; MARS Consortium. Estimated dead space fraction and the ventilatory ratio are associated with mortality in early ARDS. Ann Intensive Care. 2019;9:128. 31754866 10.1186/s13613-019-0601-0 PMC6872683

[19] Papoutsi E Giannakoulis VG Routsi C Kotanidou A Siempos II. Association between ventilatory ratio and mortality persists in patients with ARDS requiring prolonged mechanical ventilation. Intensive Care Med. 2023;49:876–7. 37258983 10.1007/s00134-023-07107-7

