# Supplementary material for: Generalized additive mixed model to evaluate the association between ventilatory ratio and mortality in patients: A retrospective cohort study
Source: Medicine (Baltimore). 2024 Nov 1;103(44):e40310. doi: 10.1097/MD.0000000000040310 (PMC11537620; doi:10.1097/MD.0000000000040310)
Supplement: Supplementary file 1 [file medi-103-e40310-s001.pdf]

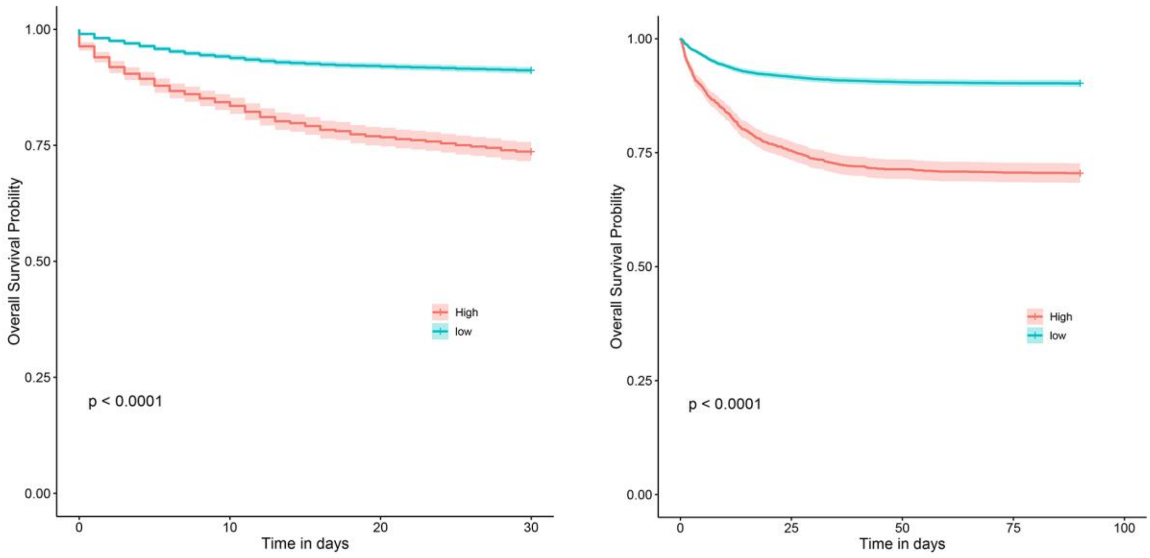

eFigure 1 Kaplan－Meier curves of ICU 30-day and 90-day mortality for the low and high VR groups

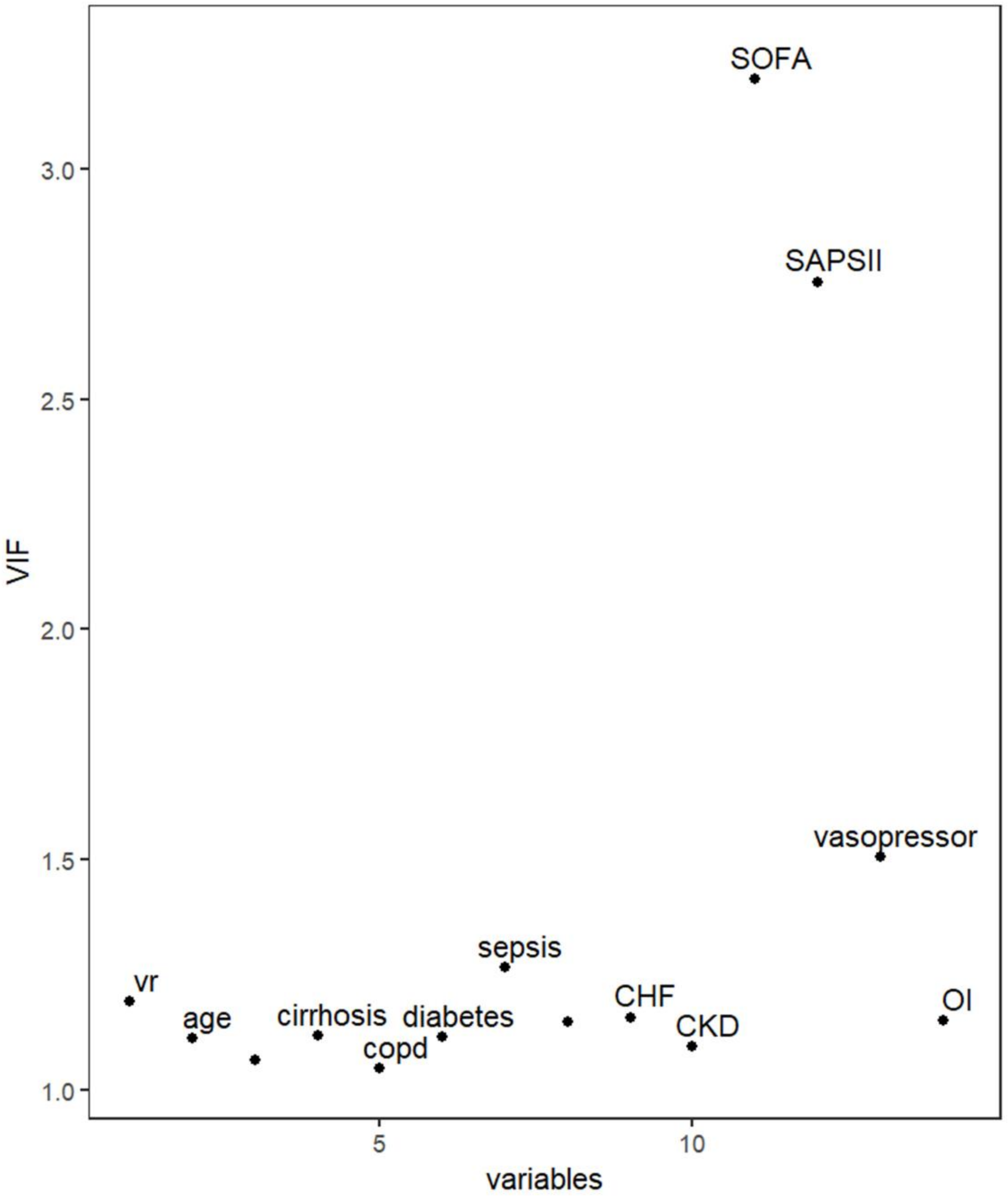

eFigure 2 Variance inflation factor(VIF) for variables contributing to 30-day mortality

Variable	Count	Percent	P value
Overall	8024	100	<0.001
Gender			NA
F	3058	38.1	<0.001
M	4966	61.9	<0.001
Age			NA
0	3854	48	<0.001
1	4170	52	<0.001
Cirrhosis			NA
0	7669	95.6	<0.001
1	355	4.4	<0.001
Diabetes			NA
0	5637	70.3	<0.001
1	2387	29.7	<0.001
Sepsis			NA
0	7077	88.2	<0.001
1	947	11.8	<0.001
COPD			NA
0	7801	97.2	<0.001
1	223	2.8	0.055
Hypertension			NA
0	3873	48.3	<0.001
1	4151	51.7	<0.001
CHF			NA
0	4322	53.9	<0.001
1	3702	46.1	<0.001
CKD			NA
0	6980	87	<0.001
1	1044	13	<0.001
ARDS			NA
0	6518	81.2	<0.001
1	1506	18.8	<0.001
SOFA			NA
0	4852	60.5	<0.001
1	3172	39.5	<0.001
SAPSII			NA
0	4086	50.9	<0.001
1	3938	49.1	<0.001
Vasopressor			NA
0	7387	92.1	<0.001
1	637	7.9	<0.001
OI			NA
<100	1335	16.7	<0.001
100-200	1336	16.7	<0.001
200-300	1016	12.7	<0.001
>300	4303	53.9	<0.001

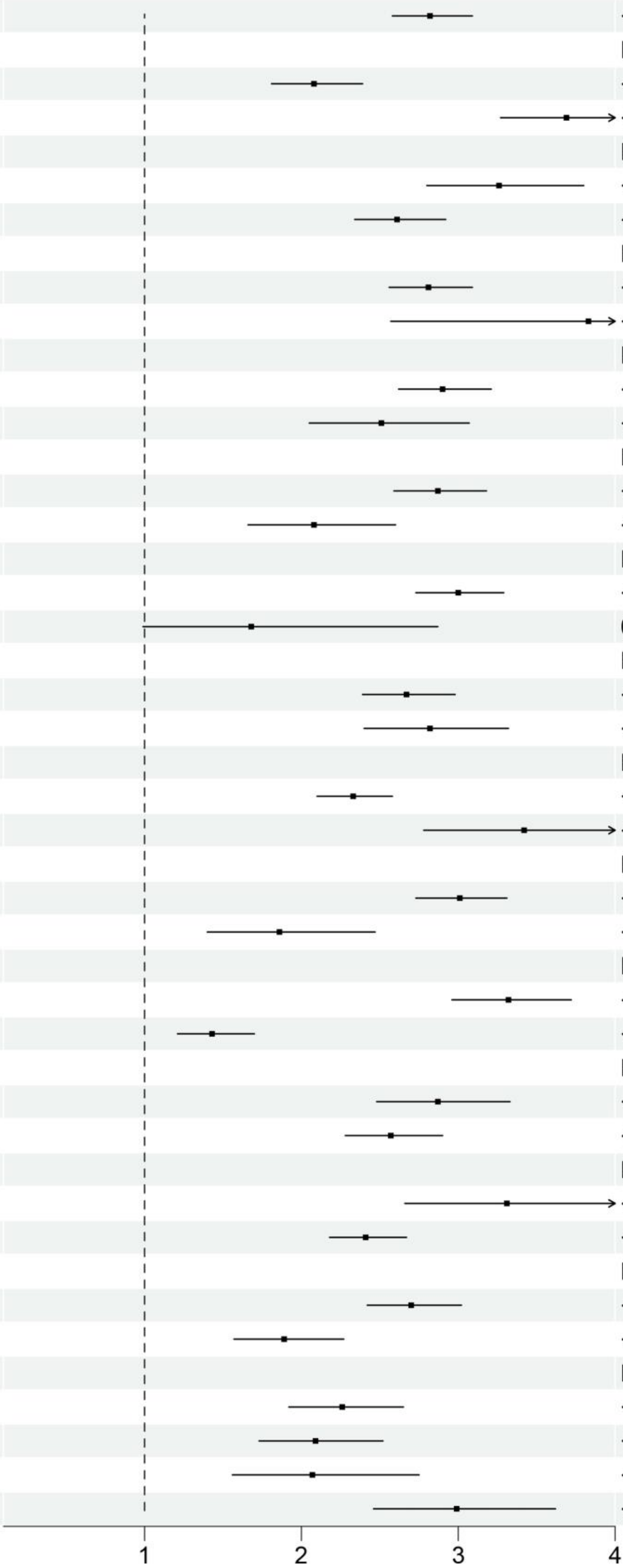

eFigure 3 Subgroup analysis of the association between VR and 30-day mortality(age was divided by 65, SOFA was divided by 3, SAPSII was divided by 36)
