# Supplementary material for: Generalized additive mixed model to evaluate the association between ventilatory ratio and mortality in patients: A retrospective cohort study
Source: Medicine (Baltimore). 2024 Nov 1;103(44):e40310. doi: 10.1097/MD.0000000000040310 (PMC11537620; doi:10.1097/MD.0000000000040310)
Supplement: Supplementary file 2 [file medi-103-e40310-s002.docx]

**eTable1**  Comparison of the changes (0-30 days) in VR between 30-day survivors and non-survivors

| **Time** | **Survivors** | **Non-survivors** | **P-value** |
| --- | --- | --- | --- |
| On admission | 1.59±0.53 | 1.94±0.70 | <0.001 |
| On 2-5th day | 1.67±0.48 | 1.81±0.61 | <0.001 |
| On 6-10th day | 1.89±0.52 | 1.99±0.61 | <0.001 |
| On 11-15th day | 1.97±0.53 | 2.11±0.67 | <0.001 |
| On 16-20th day | 1.98±0.53 | 2.28±0.72 | <0.001 |
| On 21-25th day | 2.01±0.54 | 2.33±0.77 | <0.001 |
| On 26-30th day | 2.03±0.55 | 2.45±0.86 | <0.001 |
